# Assessing the Human Health Benefits of Climate Mitigation, Pollution Prevention, and Biodiversity Preservation

**DOI:** 10.5334/aogh.4161

**Published:** 2024-01-05

**Authors:** Philip J. Landrigan, Michael Britt, Samantha Fisher, Amelia Holmes, Manasi Kumar, Jenna Mu, Isabella Rizzo, Anna Sather, Aroub Yousuf, Pushpam Kumar

**Affiliations:** 1 Global Observatory on Planetary Health, Boston College, Chestnut Hill, MA, US; 2 Centre Scientifique de Monaco, MC; 3 City University of New York, Graduate School of Public Health and Health Policy, New York City, NY, US; 4 The Lifescape Project, UK; 5 Department of Psychiatry, University of Nairobi, Kenya; 6 Institute for Excellence in Health Equity, New York University Grossman School of Medicine, New York, US; 7 The George Washington University, Elliot School of International Affairs, Washington D.C., US; 8 Harvard Medical School, Boston, MA, US; 9 United Nations Environment Programme, Nairobi, KE

**Keywords:** climate change, pollution, biodiversity loss, Triple Planetary Crisis, Global Burden of Disease (GBD) study, co-benefits

## Abstract

**Background:**

Since the Industrial Revolution, humanity has amassed great wealth and achieved unprecedented material prosperity. These advances have come, however, at great cost to the planet. They are guided by an economic model that focuses almost exclusively on short-term gain, while ignoring natural capital and human capital. They have relied on the combustion of vast quantities of fossil fuels, massive consumption of the earth’s resources, and production and environmental release of enormous quantities of chemicals, pesticides, fertilizers, and plastics. They have caused climate change, pollution, and biodiversity loss, the “Triple Planetary Crisis”. They are responsible for more than 9 million premature deaths per year and for widespread disease – impacts that fall disproportionately upon the poor and the vulnerable.

**Goals:**

To map the human health impacts of climate change, pollution, and biodiversity loss. To outline a framework for assessing the health benefits of interventions against these threats.

**Findings:**

Actions taken by national governments and international agencies to mitigate climate change, pollution, and biodiversity loss can improve health, prevent disease, save lives, and enhance human well-being. Yet assessment of health benefits is largely absent from evaluations of environmental remediation programs. This represents a lost opportunity to quantify the full benefits of environmental remediation and to educate policy makers and the public.

**Recommendations:**

We recommend that national governments and international agencies implementing interventions against climate change, pollution, and biodiversity loss develop metrics and strategies for quantifying the health benefits of these interventions. We recommend that they deploy these tools in parallel with assessments of ecologic and economic benefits. Health metrics developed by the Global Burden of Disease (GBD) study may provide a useful starting point.

Incorporation of health metrics into assessments of environmental restoration will require building transdisciplinary collaborations. Environmental scientists and engineers will need to work with health scientists to establish evaluation systems that link environmental and economic data with health data. Such systems will assist international agencies as well as national and local governments in prioritizing environmental interventions.

## Introduction

Human health and the health of planet earth – our Common Home – are inextricably linked [1]. All humans depend on the planet’s resources for food, fuel, shelter, drinking water, breathable air, a temperate climate, and mental and spiritual sustenance [2]. The global economy also relies on the planet. An estimated two-thirds of the world’s economic activity depends directly or indirectly on resources and services provided by the planetary environment [3]. Harms done to the health of the planet ultimately harm human health, and actions taken to protect the planet by mitigating climate change, preventing pollution, and preserving biological diversity benefit human health and well-being.

Humans of our species, *Homo sapiens*, have inhabited the earth for an estimated 100,000–200,000 years, but it is only in the 11,000 years since the last Ice Age – the Holocene era – that humans have prospered. This unique flourishing is directly attributable to the highly favorable environmental conditions that have characterized this era. The most unique feature of the Holocene has been a temperate climate that has been stably sustained across many millennia and allowed humans to move beyond the exigencies of day-to-day survival and cultivate crops, domesticate animals, build cities, and develop civilizations [4].

The favorable conditions of the Holocene and the agrarian, the industrial, and most recently, the technological revolutions have produced unprecedented gains. Humans have amassed unprecedented wealth. We have created art, composed symphonies, decoded the genome, explored the farthest reaches of the planet, and ventured into space. We have reduced poverty. The proportion of people living in extreme poverty has fallen from 63% of the global population in 1950 to 10% in 2017, today, despite growth of the global population from 2.5 billion to 7.3 billion [5]. We have improved health. Life expectancy in high-income countries has increased from 40–50 years in 1900, to more than 80 years today. Child mortality in almost all nations has fallen dramatically [6].

However, humanity has made these advances at great cost to the planet. We have recklessly consumed the earth’s resources and given little thought to the consequences. We have burned massive quantities of fossil fuels. We have released great and growing quantities of chemicals, pesticides, fertilizers, and plastics – many of them toxic – into the earth’s environment. We have destroyed habitats and greatly accelerated the extinction of living species.

To provide a framework for mapping anthropogenic damage to the planet’s life-support systems, Rockström et al. developed the concept of Planetary Boundaries [7]. They argue that human influence on the planetary environment is at or near the point of transgressing several of the boundaries that define a safe operating space for humanity. For the 11,000 years of the Holocene Era, these support systems have operated within safe boundaries. They have maintained a temperate climate, provided clean breathable air, recycled nutrients such as nitrogen and phosphorus, and regulated the world’s water cycle, providing freshwater for drinking and sanitation [7]. Now, the boundaries for several planetary support systems – climate, biodiversity, the nitrogen cycle, and pollution by synthetic chemicals and plastics – appear to have been transgressed. We may be approaching a point where the damage can no longer be reversed [8].

## Methods

To map the physical and mental health impacts of current planetary changes, we conducted a scoping review using the search terms “Climate Change”, “Pollution”, “Biodiversity Loss”, and “Triple Planetary Threat” with particular emphasis on review articles and publications by international agencies that linked these threats to physical and/or mental health.

To examine the potential benefits for physical and mental health of investments in climate mitigation, pollution prevention, and preservation of biological diversity, we studied selected flagship projects implemented by the Global Environmental Facility (GEF), Green Climate Fund (GCF) and the UN Environment Programme (UNEP).

## Findings

### Health Effects of Climate Change

Climate change poses grave threats to human health and to the stability of modern societies [9]. The main driver is a sharp increase in emissions of carbon dioxide (CO_2_) and other greenhouse gases resulting from massive combustion of fossil fuels. Land-use changes such as deforestation place additional pressures on the climate by reducing the capacity of ecosystems to absorb and store atmospheric carbon dioxide [1].

With climate change, the mean temperature of the earth’s surface has warmed by approximately 1.2 degrees centigrade since 1880, and the rate of increase has accelerated since 1970. Sixteen of the 17 world’s warmest years have occurred since 2000 [9]. This increase in mean surface temperature is not evenly distributed. In some parts of the world, temperature has increased little or not at all, but other places, especially the circumpolar regions, have experienced increases as great as 2–3 degrees Celsius [10,11].

#### Extreme Weather Events and Health

Climate change resulted in increased frequency of severe heat waves and heat-related illness. It has increased the frequency and severity of extreme weather events including hurricanes and cyclones of enormous destructive capacity, all of which cause great harm to human health [12,13,14].

#### Flooding and Health

Climate-related flooding occurs across a range of environments, including mountainous regions, where it is the consequence of fluvial flooding from heavy rainfall and snowmelt, and coastal regions where storm surges, tsunamis, and high tides cause damage, especially ink major port cities, and increase disease risks [15,16,17].

Diverse, healthy ecosystems ameliorate the intensity of floods. Floodplains and wetlands provide spaces to retain water surplus, decreasing the severity of flooding. Forests provide natural flood hazard mitigation by absorbing large amounts of water from rainfall, thus reducing surface runoff and river discharge. Forests reduce wind velocity, a contributing factor to snow melt. Mangrove forests protect coastlines [18].

With global climate change, flooding will increase in frequency and intensity. Anthropogenic land-use change will further exacerbate these effects. All of these impacts are intensified by poverty and are most harmful in LMICs [19].

#### Vector-Borne Diseases and Health

Climate change increases risk of infection by vector-borne diseases [20,21], including diseases carried by mosquitoes (malaria, West Nile virus, and yellow fever) [22] and ticks (Lyme disease) [23]. Many temperate regions are beginning to see increasing incidence rates of diseases previously confined to warmer regions (e.g., malaria in Europe, dengue in the southern United States, and poleward expansion of Lyme disease).

#### Water Scarcity and Health

Climate change and loss of natural ecosystems exacerbate water scarcity. Water scarcity is further aggravated by unsustainable water extraction for agriculture across vast areas of China, India, and the United States. The potential consequences include food shortages, social unrest, migration, and conflict [24].

### Health Effects of Pollution

Pollution is the largest environmental cause of disease and premature death in the world today [25]. It arises from the same sources that drive climate change. It was responsible in 2019 for an estimated 9 million deaths worldwide [26,27], including one million deaths in children [28]. In many regions, ambient air pollution and chemical pollution, the forms of pollution most closely associated with urbanization and modern industrial development are worsening.

#### Air Pollution and Health

**Figure 1:**
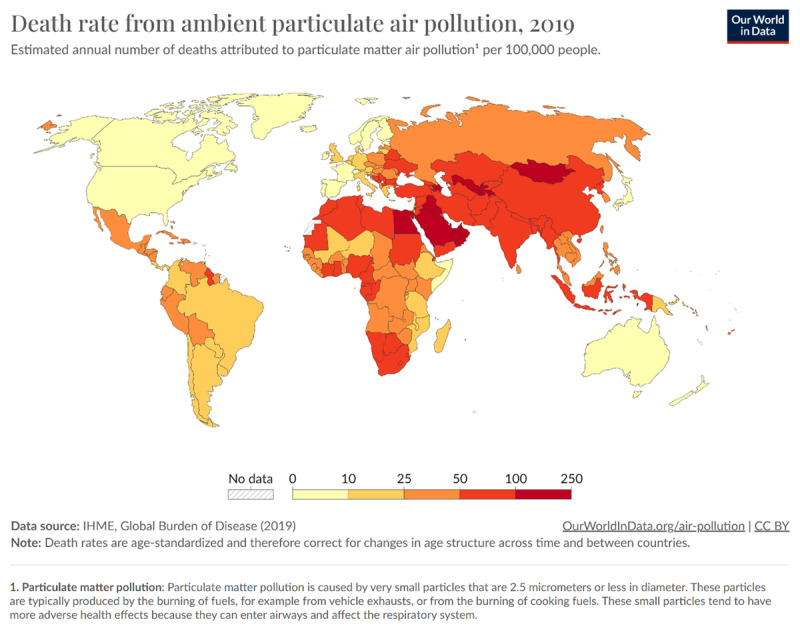
Death rate from ambient particulate air pollution by country, 2019. Source: IHME [14].

The Global Burden of Disease (GBD) study estimates that, in 2019, ambient air pollution was responsible for approximately 7 million deaths worldwide [27,29]. Ambient air pollution is worsening as climate change progresses. The number of deaths attributable to ambient air pollution has increased by 51% since 1990 and continues to rise [30]. Sharpest increases are seen in the rapidly growing cities of low- and middle-income countries [31]. (Figure 1) Without aggressive intervention, the number of deaths attributable to ambient air pollution could double by 2050 [32].

Climate change and air pollution are intimately linked [26]. Fuel combustion – fossil fuel combustion in high-income and middle-income countries and biomass burning in low income countries – is responsible for 80% of the greenhouse gases and short-lived climate pollutants that drive climate change. Additionally, fuel combustion creates 85% of airborne particulate pollution and almost all air pollution is associated with sulfur and nitrogen oxides (SO_X_ and NO_X_) [26].

#### Chemical and Plastic Pollution and Health

Chemical and plastic pollution is a great and growing global problem [27]. More than 325,000 new chemicals and chemical mixtures have been synthesized since 1950 [33]. At the same time, more than 8 Gigatons of plastic have been produced [34]. More than 98% of these materials are produced from coal, oil, and gas. Production of petrochemicals and plastics is an important driver of both climate change and air pollution.

Synthetic chemicals and plastics have become widely dispersed in the global environment. They are responsible for nearly universal human exposure and widespread disease [34].

#### Water Pollution and Health

In 2015, an estimated 1.3 million deaths were caused by unsafe water sources [26]. Water pollution is a mix of human waste, animal waste, agricultural run-off, and chemical discharges. The principal diseases linked to water pollution are acute and chronic gastrointestinal diseases – diarrheal diseases (70% of deaths), typhoid fever (8%), paratyphoid fever (20%), and lower respiratory tract infections (2%). Polluted water is linked, additionally, to a range of parasitic infections.

Substantial progress has been made in reducing water pollution and waterborne disease. Between 1990 and 2015, 2.6 billion people gained access to improved drinking water sources. In this time, the number of children dying from diarrheal diseases decreased from approximately 1.5 million deaths in 1990 to slightly greater than 0.6 million in 2012 [26].

#### Ocean Pollution and Health

Ocean pollution is a critically important but underrecognized component of global pollution. It has multiple direct and indirect impacts on human health. The nature and magnitude of these impacts are only beginning to be quantified [35].

Sea surface warming and worsening marine pollution result in expanding geographic ranges of marine pathogenic bacteria. The result is that bacteria such as *Vibrio* species are moving poleward into cold, previously unpolluted waters to cause previously unseen, life-threatening infections [36].

Absorption into the oceans of increasing amounts of atmospheric CO_2_ causes ocean acidification that in turn destroys coral reefs and marine microorganisms. These events contribute to reduction of fish stocks and increased risk of malnutrition, especially in coastal communities in low-income and middle-income countries [37].

### Health Effects of Biodiversity Loss

Biological diversity is a hallmark of healthy, functioning ecosystems. Healthy ecosystems support ecosystem services such as pollination, healthy soils, and fresh water that are critical to human health and the sustainability of modern societies [38].

**Figure 2:**
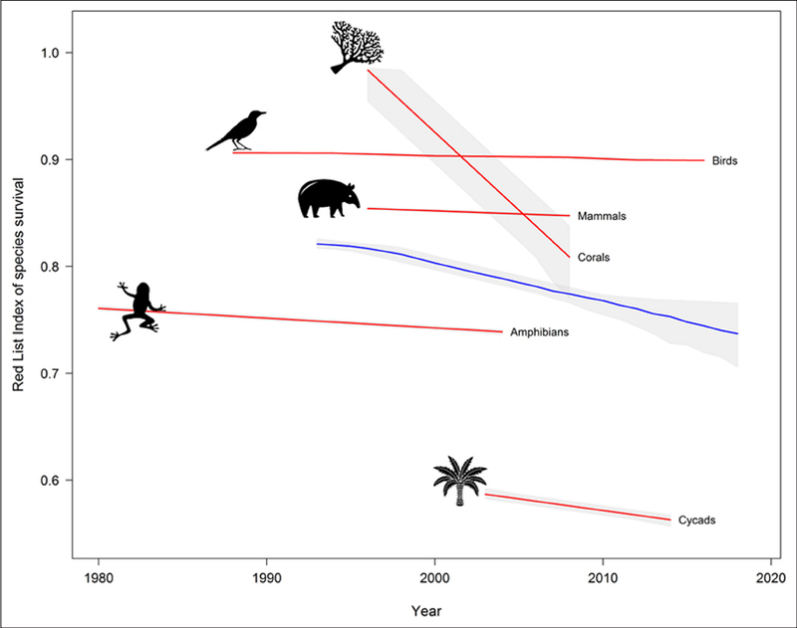
The Red List Index (RLI) of species survival for mammals, birds, amphibians, reef-forming corals and cycads. The blue line indicates the overall RLI for all the taxa combined. Coral species are moving towards increased extinction risk most rapidly, while amphibians are, on average, the most threatened animal group. Source: IUCN [41].

Climate change, pollution, land-use change, and habitat destruction all threaten biodiversity. These pressures have greatly accelerated the rate of species extinction, which is estimated to be 100-1000 times greater today than a millennium ago [39] and is projected to worsen still further over the next 50 years [40]. Species currently facing extinction include 12% of all birds, 32% of amphibians, 23% of mammals, 31% of gymnosperms, and 33% of corals (of those assessed by the IUCN Red List) [41,42,43] (Figure 2).

Accelerating biodiversity loss destabilizes ecosystems, reduces ecosystem productivity, impedes the ability of ecosystems to decompose waste and recycle biologically essential nutrients. Heightened risks of disease and death are among the consequences [44,45,46,47].

#### Biodiversity, Food Production, and Health

Global food security is highly dependent on rich biological diversity [48]. Healthy ecosystems provide humans with food and nutrition from multiple sources that include highly managed systems such as crops, aquaculture, and livestock as well as wild sources such as capture fisheries, game and wild plants [49].

#### Biodiversity, Soil Degradation, and Health

Modern chemically intensive, commodity-oriented agriculture has diminished soil quality in many places, thus reducing soil fertility [50,51]. It has also increased soil loss through wind and water erosion faster than it can be replenished. Loss of between 1 and 12 million hectares of agricultural land is occurring annually [52]. In many regions, nutrient depletion of soils has led to declines in agricultural output [53].

#### Biodiversity, Agrochemical Inputs, and Health

Nitrogen- and phosphorus-containing fertilizers are produced in massive quantities and used extensively in agriculture [50]. Wide use of these chemical fertilizers has led to accumulations of phosphorus and nitrogen in aquatic ecosystems, with resulting eutrophication, harmful algal blooms, and fish ‘die-offs’ [54,55,56].

Pesticides further damage ecosystems and contribute to biodiversity loss [57,58]. Pesticides also directly harm human health resulting each year in thousands of acute poisoning episodes [59] as well as reduced male fertility [60], birth defects [61], cancers, and respiratory disease [62].

Traditional and integrative agricultural systems, which lessen dependence on agrochemicals, may be expected to decrease incidence of pesticide-related diseases, while at the same time enhancing soil quality, increasing crop yields, and reducing farmers’ out-of-pocket costs [63,64]. An example is seen in the scaling up of Zero Budget Natural Farming in India [65].

#### Biodiversity, Pollination, and Health

Insect pollination is essential for the reproductive cycles of over 87 of the leading global food crops [66]. Honeybees alone pollinate over 20,000 plant species and butterflies, flies, moths, wasps, beetles, birds, bats, and other animals that contribute further. Pollinators are critical for horticulture, orchard, and forage production [67]. Fiber and root crops rely on pollinators for the production of seed [68]. Approximately 80% of all flowering plant species, and more than 75% of global food crops, including fruits and vegetables and some of the most important cash crops, such as coffee, cocoa and almonds, rely on animal pollination.

Reductions are reported in numbers and diversity of both wild and domestic pollinators [69]. Loss of pollinating insects poses a grave threat to food production and thus to global food security. Neonicotinoid insecticides are strongly implicated in pollinator decreases [70]. Reductions in honeybee populations can lead to increased exposure of food crops to pests and parasites, reduced genetic diversity and other environmental stressors [71,72,73].

#### Biodiversity, Pollution Control, and Health

Healthy ecosystems reduce pollution by absorbing, processing, sequestering, and detoxifying wastes, thus reducing the concentrations of harmful substances that threaten human health [74].

Vegetation in urban areas plays an essential role in regulating air quality by mitigating particulate pollutants such as dust, ash, pollen, and smoke as well as absorbing toxic gases like ozone, sulfur dioxide, and nitrogen dioxide [75]. Trees and forests in the conterminous US removed an estimated 17.4 million tons of pollution from the air in 2010 [76]. Natural ecosystems can mitigate temperature extremes in both warm and cold seasons and in turn reduce pollution emissions from power-generating facilities [77].

Water purification is an ecosystem service provided by forests, wetlands, and grasslands, which slow the movement of water from source to destination and thus filter the water [78]. Healthy ecosystems containing diverse microbial assemblages provide diverse purification services that include detoxifying pesticides and removing heavy metals [79].

#### Ocean Biodiversity and Health

Global food security is dependent on the health of fisheries. An estimated 3 billion people obtain 20% or more of their protein intake from fish [80]. Due to overfishing, global warming, marine habitat degradation, ocean pollution, and ocean acidification, fish stocks are declining worldwide and multiple fisheries are in jeopardy [81].

#### Biodiversity and Medicine

Healthy functioning ecosystems provide many medicines and chemical compounds used by humans [40], and more than 60,000 species of plants, animals, fungi and microbes are utilized to make medicines and other beneficial products [2]. An estimated 4 billion people rely primarily on natural medicines for their health care [82,83]. Higher levels of biodiversity in natural ecosystems increase the probability of discovering new natural compounds with potential medicinal uses [84,85].

The International Union for Conservation of Nature red list indicates that more than 13% of the 5,000 currently known medicinal plants are classified as under threat [41]. Unless urgent action is taken to protect biological diversity, more species will be lost, and the genetic and biological secrets these organisms hold will be gone forever.

#### Biodiversity and Disease Control

Zoonoses are a growing challenge to human health [46]. They include diseases caused by bacteria, fungi, parasites, viruses, and prions [86]. An estimated 60% of emerging infectious diseases are zoonotic in origin, including Ebola, Zika and Nipah encephalitis and almost all known pandemics such as influenza [87], HIV/AIDS and more recently COVID-19 [88]. Annually around 2.7 million deaths are attributed to zoonotic infections [89].

**Figure 3:**
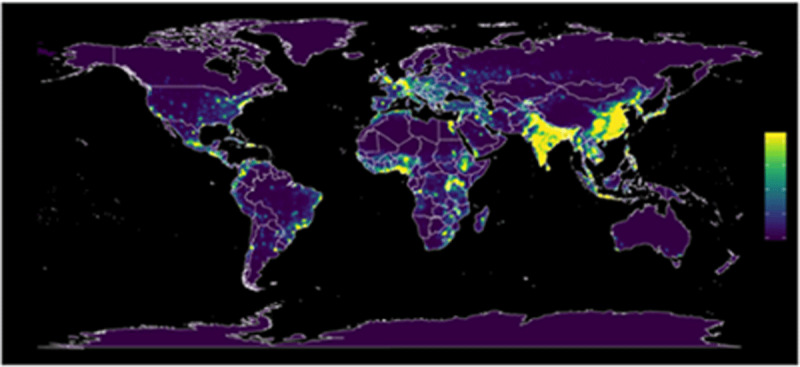
Estimated risk of zoonotic emerging infectious disease events. Source: (Allen et al., 2017) [95]. **License:** https://creativecommons.org/licenses/by/4.0/legalcode

The risk of novel zoonotic disease outbreaks is increasing, owing to anthropogenic environmental changes [90]. Human activities such as land use change (logging, mining) and monoculture agriculture that increase human contact with previously sequestered species and increase the frequency of zoonotic “spillover” events [91] (Figure 3). Over 50 per cent of the emerging infections of zoonotic origin between 1940 and 2000 are attributed to human drivers [46,92,93].

With ecosystem destruction and loss of wild habitats, species that act as buffers against disease transmission may be reduced in number or even become locally extinct. This loss of biodiversity is especially problematic for human health when the species lost are those that effectively “sequester” infectious diseases due to their extremely robust immune systems and low rates of reproduction. With increased ecosystem disruption, species with high fecundity and weaker immunity can move in to occupy niches previously filled by buffering species.

The concept that high ecosystem biodiversity results in a lower abundance of competent hosts and higher abundance of less competent hosts connects biodiversity with a decreased risk of epidemics, and is called the “dilution effect hypothesis” [93].

**Box: Dilution Effect of Biodiversity; Case Study on Lyme disease**

Lyme Disease (caused by *B. burgdorferi*) in Northeastern and Midwestern North America is spread primarily via the blacklegged tick. Biodiversity has been observed to reduce transmission of Lyme disease to humans.

Schmidt and Ostfeld [94] used empirical and modeling approaches to measure the dilution effect across New York State. They found that as species richness increased, the prevalence of Lyme disease in field-collected ticks decreased. They concluded that an increase in species richness reduces risk of Lyme disease transmission, providing evidence for a dilution effect. Similar patterns have been observed in Europe.

**Figure 4:**
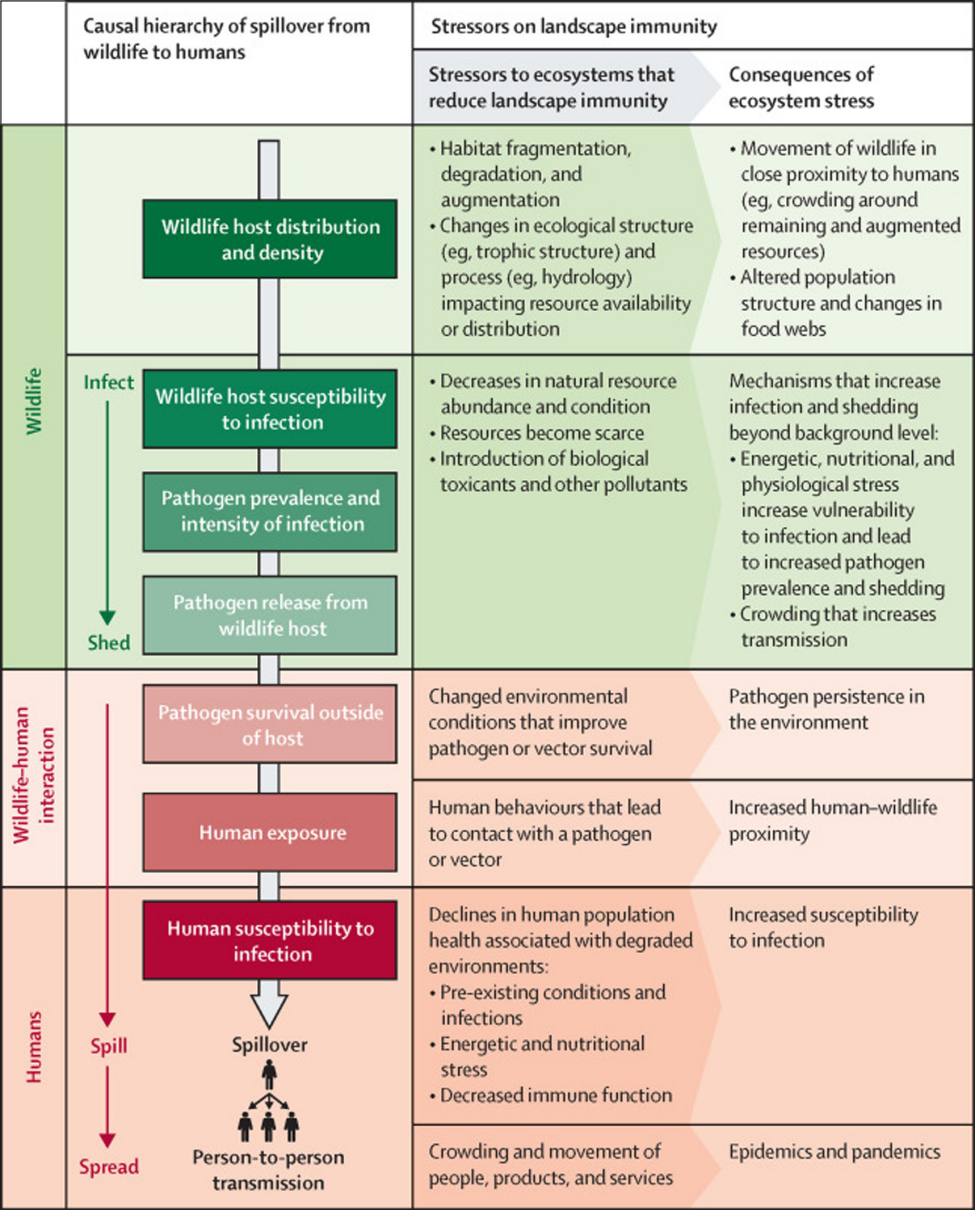
Land-use induced spillover of zoonotic infections. Source: Plowright et al. [46].

Efforts aimed at early detection and response to emerging pathogens have gained traction in recent years, particularly given the ongoing devastation caused by the COVID-19 pandemic. Emerging infectious disease modeling has assessed global distribution of emerging infectious disease risk (Figure 4). Regions identified as having an elevated risk for disease emergence are tropical regions, particularly those undergoing rapid encroachment of human settlement and agriculture into previously intact forest ecosystems [95].

### Mental Health Effects of Climate Change, Pollution, and Biodiversity Loss

Healthy, non-polluted ecosystems provide multiple benefits to human well-being and mental health including aesthetic enjoyment of nature, recreation, spiritual experiences, and physical and mental health benefits [96]. Activities such as recreation and ecotourism allow people to immerse in the natural environment through camping, hiking, and nature study. For many communities and cultures, nature also holds significant spiritual and religious value.

Conversely, environmental disruptions such as climate change, pollution, and biodiversity loss have powerfully negative impacts on mental health [97,98]. These impacts are particularly prevalent in LMICs that are experiencing rapid epidemiological and demographic changes, such as the rise of megacities. They are exacerbated by food and water scarcity, diminished income, housing insecurity and inadequate healthcare systems and further aggravated by uncontrolled population growth, shifting demographics, urbanization, and greater consumption of natural resources.

#### Mental Health Effects of Climate Change

Climate change negatively affects mental health through numerous pathways including exposure to extreme heat and high temperatures, extreme weather-related events, and natural disasters [98]. Exposure to extreme heat and high temperatures are associated with increased deaths by suicide and increased admissions for inpatient psychiatric treatment. Heat waves are associated also with increased aggression [99]. Droughts, floods, and violent storms are associated with depression, anxiety, psychological distress, and suicide [100,101,102,103].

#### Mental Health Effects of Pollution

Air pollution is known to increase the risk of new onset of mental disorders and to exacerbate the severity of existing mental disorders [104]. A series of panel studies in China found that air pollution is associated with an increase in severe mental illness as well as with nearly $23 billion in health expenditures due to treatment [105].

Pollution by toxic metals and manufactured chemicals is a powerful and preventable cause of brain damage and mental disorder. Early-life exposures to pollutants have been linked to decreased cognitive function, reduced IQ, shortened attention span, and increased risk for attention-deficit hyperactivity disorder (ADHD) [106].

Chemical pollution and air pollution tend to concentrate in poor, minority, and marginalized communities and in LMICs – a classic example of environmental injustice – and can magnify the negative impacts of social disadvantage on mental health [107].

Lack of green spaces, particularly in urban built environments, has been shown to be associated with depression, anxiety, and stress. Conversely, the inclusion of green spaces in the living environment improves mood and well-being [108]. Green spaces include urban forests, “green design” buildings, green exercise space, and water bodies or “blue spaces” [109,110,111].

Noise pollution includes road noise, traffic noise, and ambient noise exposure. Noise pollution is associated with hyperactivity and inattention, irritability, anxiety symptoms, and depression [112,113].

## Assessing Flagship UN Projects and Their Health and Environmental Benefits

To examine the strategies used by international agencies and national governments for assessing the health benefits of their investments in climate change mitigation, pollution prevention, and preservation of biological diversity, we examined flagship projects implemented by UNEP through the Global Environment Fund (GEF) and the Green Climate Fund (GCF). Additionally, we examined the Economics of Ecosystems and Biodiversity (TEEB) program hosted by UNEP.

The goal of these analyses was to determine what strategies are currently used by international agencies for assessing the health benefits of environmental interventions to see what works, to understand could be upscaled, and to identify gaps.

### The Green Climate Fund (GCF)

The Green Climate Fund (GCF) [114] is the financial arm of the UN Framework Convention on Climate Change and is supported by the 194 countries that are Parties to this Convention. GCF invests in low-emission and climate-resilient development. It focuses especially on regions highly vulnerable to climate change, including Least Developed Countries (LDCs), Small Island Developing States (SIDS), and various African states.

GCF strives to maximize its impact by diversifying its portfolio equally across both climate adaptation and climate mitigation. It engages with both public and private financial sectors. It provides a wide variety of financial products. It acknowledges the importance of developing nations’ integrating GCF funds into their own national development plans. Since its founding in 2010, GCF has funded 143 projects and committed $6.2 billion to these investments.

### The Global Environment Fund (GEF)

The Global Environment Fund (GEF) [115] is a private equity fund that invests in corporations to promote green energy and sustainable use of the earth’s resources within the corporate sphere. GEF operates on the premise that a sustainable and environmentally centered approach to finance holds the potential for an improved environment, economy, and quality of life.

GEF partners with companies that offer environmentally friendly products. Through an Operational Due Diligence Process, it selects companies that fit their criteria of having a stable, proven, and upward trajectory of revenue as well as having a high likelihood of improving the global environment. To date, GEF has invested nearly $1.0 in environmentally friendly and efficient energy and resource companies.

### The Economics of Ecosystems and Biodiversity (TEEB)

TEEB is a global program funded by the International Climate Initiative and hosted by UNEP [116]. TEEB is focused on “making nature’s values visible”. Its objective is to incorporate valuations of biodiversity and ecosystem services into decision-making at all levels. TEEB has conducted projects in many countries, including Columbia, Kenya, Tanzania, and Thailand. It utilizes a three-step approach to valuing biodiversity and ecosystem services:

- **Recognizing cultural and spiritual value in ecosystems**, including landscapes and species. For example, the protection of sacred groves in some countries has helped to protect these special areas and the biodiversity they contain.
- **Quantifying the economic value of ecosystems**. This information enables decision makers to consider the full benefits of nature and the potential costs of lost ecosystem services. It thus moves decision making beyond a narrow focus on the value of produced goods and GDP. An example is calculation of the economic value of the flood control ecosystem services provided by wetlands, salt marshes, and mangrove forests compared to the costs of destroying these natural systems and replacing them with concrete flood barriers.
- **Introducing the economic values of ecosystems and biodiversity into decision making**. This can include providing incentives for preservation of ecosystem services, eliminating environmentally harmful subsidies, or introducing tax breaks for conservation.

Advancement of Natural Capital Accounting is a major TEEB initiative. Unlike GDP, which examines only the value of goods and services produced by human activity, Natural Capital Accounting seeks to develop a broader and more comprehensive valuation of national wealth that includes assessments of the economic worth of natural resources and ecosystem services. An example is quantification of the economic value of the air filtration, carbon sequestration, flood control and climate modulation produced by forests.

Another TEEB initiative, Supporting Biodiversity and Climate-Friendly Land Management in Agricultural Landscapes, applies systems thinking to the economics of agriculture. It identifies and quantifies the huge but hidden and externalized costs of modern agricultural systems.

This project has identified agricultural practices that benefit the environment and human health, developed metrics and techniques to assess the economic value of these practices, and used this information to leverage policy changes and legislative reforms. UNEP is currently supporting the implementation of TEEB AgriFood initiatives in 10 countries, in collaboration with national and local government agencies, and local research institutions.

The following are case studies that highlight some of UNEP’s work in protecting human health by safeguarding the earth’s environment.

**Case Study 1. Developing Core Capacity to Address Adaptation to Climate Change in Productive Coastal Zones in Tanzania [117]**

Tanzania’s coastline is vital to the country’s economy. Rising sea levels resulting from global climate change pose a significant threat to coastal and island communities as well as to biological diversity. Without action, rising sea level is projected to cost the Tanzanian economy upwards of $200 million per year in the next 30 years and the resulting floods threaten $5.3 billion in assets.

In collaboration with the Division of Environment of the Vice President’s Office of Tanzania, UNEP, Global Environment Fund, and the Adaptation Fund, various stakeholders have worked to implement ecosystem-based adaptations throughout major population centers along the Tanzanian coast, including the rapidly expanding metropolis of Dar es Salaam. To combat the effects of rising sea levels, officials rooted their efforts in natural and structural improvements. For example, numerous seawalls, groynes, and dikes were built to limit the erosion and structural damage caused by the sea. Officials also restored local coral and mangrove habitats, which have been proven to protect against storm surges. Mangrove habitats were designated as “no-take zones” in order to reduce deforestation. Community initiatives were established, including a network of 87 local groups tasked with managing and protecting the regrowing mangrove sites. In addition, approximately 3,000 efficient stoves were distributed to households in areas that have traditionally relied on mangroves for fuel.

This initiative has proven highly successful, and it has benefited the Tanzanian environment and nearly one million Tanzanians, both directly and indirectly. Furthermore, 3,000 m^2^ of coral reefs and 1,000 ha of mangrove forest have been restored. Community members were trained in maintaining these resources, with at least 100 people being trained in coastal and climate vulnerability mapping techniques. Through this work, UNEP and partner associations are fostering a culture of sustainable and intergenerational change, and by providing necessary skills and building capacity in individuals and local communities, this change is likely to be long-lasting and sustainable.

Excellent strides have been made in restoring natural resources that mitigate climate change and protect against sea level rise, while advancements in infrastructure contributed further to protection against environmental disasters. These efforts also reduce risk of death and injury from environmental disasters in the areas where interventions have been successfully implemented.

A detailed analysis of this initiative revealed certain limitations and areas for future improvement. According to the *UN Environment GEF PIR Fiscal Year 2019* project document, the efficiency and success of this project were slightly hindered by a small overall budget in comparison to the magnitude of the project’s outputs and by a delay in the Tanzanian audit procedure. However, the project successfully overcame these challenges and remained on track for financial closure. There were also some cultural and community challenges with certain community associations advocating for advance payments to manage the mangroves and by a lack of female participation in the project due to cultural norms on the Tanzanian coast that women do not participate in outdoor activities. Lastly, due to a budget cut, the number of boreholes constructed in a specific region was reduced from 17 to 10, and a road flood delayed completion of a portion of the work.

This careful review provided a series of suggestions to improve the quality of the outputs for future UNEP projects. Specifically, this analysis noted the need for improvements in the thoroughness of the vulnerability assessments, for more coastal-oriented GIS training for district officers, and for greater use of already implemented district software.

**Case Study 2. Large-Scale Ecosystem-Based Adaptation in The Gambia: Developing a Climate-Resilient, Natural-Resource-based Economy [118]**

UNEP has partnered with the Ministry of Environment, Climate Change, and Natural Resources of The Gambia to address the implications of climate change on local communities, taking an individualized approach.

One-third of the land mass of The Gambia lies less than 10 meters above sea level, and about 20% of this land is flooded seasonally. Thus, large areas of the country are at high risk for rising sea levels. To develop novel strategies to prevent this situation from worsening, officials plan to restore degraded forests, develop ecologically sustainable businesses, and create home and community gardens that would diversify food sources. A further challenge is that climate change is projected to bring erratic rainfall and droughts, which could greatly damage the economy of The Gambia’s agricultural sector, resulting in lower crop yields and a “hunger season” extending throughout the summer months.

To execute these plans, officials planted multi-purpose plant species that provided value to the economy and also augmented climate resilience. Additionally, enrichment planting was undertaken to reduce the impacts of soil erosion. Mangrove areas were established as buffer zones along the coast to decrea*s*e the impact of storm surges. Climate-resilient, natural-resource-based businesses were established to help foster the growth of the economy, while providing local residences with herbs, shrubs, and trees.

As a result of these actions, 13,400 hectares of previously degraded forests, farmland, and wildlife areas were revitalized, 46,200 households directly benefited, and 166 natural-resource-based businesses were established. The combination of environmental, economic, and cultural cultivation improvements was crucial in the long-term success of this program. To date, this is the largest natural resource development project ever undertaken in The Gambia.

Furthermore, this drastic revitalization of natural resources accomplished through this project has benefited human health and well-being and has enhanced the quality of life for many Gambians. This project has reduced the percentage of the population suffering from hunger due to increased quantity and variety of food sources. The project has also lowered anxiety levels stemming from concerns about food insecurity and thus has produced a mental health benefit that complements the improvements in physical health.

**Case Study 3. Enhancing Climate Change Resilience of Rural Communities Living in Protected Areas of Cambodia [119]**

A striking feature of the Cambodian landscape is the Mekong River, along whose banks approximately 80% of the population of this Southeast Asian nation resides. The low-lying central plains that surround the river are highly dependent on rainfall for agricultural production. As a result of climate change, rainfall has become erratic, and the frequency of torrential rains has increased. This has led to erosion, crop failures, and damaged infrastructure. To deal with the loss of income resulting from decreased agricultural production, locals have engaged in illegal logging. This has reduced tree cover, which has resulted in decreased rain cloud cover and further increased rainfall and flooding.

Cambodian officials determined that the best strategy for approaching these issues would be to reforest natural land, halt illegal logging, create home gardens, and implement an early warning climate system to guide farmers’ decisions on planting and harvesting.

To accomplish these goals, build climate adaptation and strengthen ecosystems, multi-use native tree species were planted in heavily deforested areas. New varieties of crop seeds were distributed to farmers, such as drought-resistant rice strains, and the planting of traditional crops was encouraged. At the same time, home gardens were established containing a wide array of vegetables and other sources of nutrition such as chicken and cricket coops. Finally, community patrol groups were organized to work actively against illegal logging and to protect farmers’ livelihoods.

As a result of these initiatives, 1,875 hectares of community forests were improved and restored. This has improved air quality because the newly planted trees absorb air pollutants and produce more oxygen. Over 1,900 families benefited from increased agricultural yields, and 80% of these 1,900 families reported that they had increased access to safe drinking water. By the conclusion of this project in 2019, more than 900,000 trees had been planted. This project is estimated to have produced a 20% decrease in the climate change vulnerability index across all intervention sites in Cambodia, directly benefited approximately 10,000 people, and indirectly benefited many more.

This case study demonstrates that strategies designed to increase climate resilience and reduce soil erosion in high-risk communities can also increase access to safe drinking water, restore natural crops, improve nutrition, reduce food insecurity, strengthen local economies and improve human physical health and mental well-being.

**Case Study 4. The Great Green Wall: Implementation Status and Way Ahead to 2030 [120]**

The Great Green Wall Initiative (GGWI) is a massive, African-led multinational effort to establish a “green barrier” to combat the effects of desertification and land degradation and combat drought across Africa from East to West along the southern edge of the Sahara Desert. The GGWI was endorsed and initiated in 2007 through a partnership formed by political leaders of the Sahara and Sahel regions of Africa, including Senegal, Burkina Faso, Nigeria, Sudan, and Egypt. The Great Green Wall will ultimately span 8,000 km across the African continent, with interventions occurring in 15 km units.

Trees are being planted along the GGWI line, which is already 15% complete. As these trees grow, ecosystems are flourishing, climate change mitigation and adaptation are occurring, oxygen is being produced through photosynthesis, soil erosion is diminishing, habitats are being revitalized, and food and water security are improving in local communities. The ultimate goals of the Great Green Wall Initiative are to reduce poverty and to empower local Sahara and Sahel communities to manage the land by harnessing the power of natural resources. Business partners, including the eco-friendly search engine Ecosia, have collaborated with various countries, such as Burkina Faso to assist in the implementation of the Great Green Wall by helping plant trees. Senegal and Ethiopia have been successful in planting 11 million trees and restoring 37 million acres of land.

Important unanswered questions as the program moves into its next decade are whether individual countries will be able to maintain their contributions and whether the trees, being used to mitigate desertification will survive.

These case studies demonstrate that the UN Environment Programme has made substantial progress in developing and deploying strategies and metrics within its GEF/GCF funded projects for measuring ecologic impacts and for quantifying the economic losses associated with ecosystem destruction, climate change and loss of biodiversity. UNEP has been a leader also in developing and deploying strategies for quantifying the economic benefits of climate change mitigation, ecosystem restoration and biodiversity preservation. The Economics of Ecosystems and Biodiversity (TEEB) program [116] lies at the heart of this endeavor. Specific examples of the methodology’s application to ecologic and economic assessment are the following:

- In Case Study #1, *Developing Core Capacity to Address Adaptation to Climate Change in Productive Coastal Zones in Tanzania* [117], mangrove and other marine plant communities are being restored to promote adaptation and climate resilience in coastal communities, where approximately 25% of the nation’s population, 75% of the nation’s industries, and 32% of the nation’s income are located. Through this initiative, 3,000 m^2^ of coral reefs and 1,000 hectares of mangrove forest have been restored. Community members have been trained in maintaining these resources, with at least 100 people being trained in coastal and climate vulnerability mapping techniques. This project strengthens marine communities, primarily mangrove, and coral habitats that provide the foundation for further biodiversity.
- In Case Study #3, *Enhancing Climate Resilience of Rural Communities Living in Protected Areas of Cambodia* [119], approximately 2,000 hectares of land have be restored, an estimated 1,900 families have benefited from increased agricultural yields and 80% of these families obtained increased access to safe drinking water. This project is estimated to have produced a 20% decrease in the climate change vulnerability index across all intervention sites in Cambodia, which in turn in turn had direct benefits for approximately 10,000 people.
- In Case Study #4, *The Great Green Wall*, being implemented in 20 different countries across Africa, the overarching goal is to reclaim and restore 100 million hectares of land across the Sahel region through planting millions of trees. To date, Senegal and Ethiopia have succeeded in planting 11 million trees and restoring 37 million acres of land. Arid land is being made fertile once again through these restoration processes, biodiversity is increasing, and food has become more plentiful [120].

Absent, however, from these presentations is systematic or standardized quantification of their benefits to human health. No attempts were made to estimate the number of deaths or the number of Disability-Adjusted Life-years [DALYs] averted by these interventions. In addition, no attempt appears to have been made to assess the economic value of the health improvements that resulted from these interventions.

The 2015 UNEP/WHO/Convention on Biological Diversity report, *Connecting Global Priorities: Biodiversity and Human Health* [2], speaks extensively about the negative impacts of environmental degradation on human health. It discusses the need to use common metrics and frameworks to measure the health benefits of biodiversity. This report notes the value of the disease, disability and death metrics developed by the Global Burden of Disease study [121].

## Conclusion

National and international agencies have made important progress under UNEP leadership in mitigating climate change, improving biodiversity, and reducing pollution through GEF- and GCF- funded projects. The UNEP team have developed sophisticated indicators through the TEEB program [116] to measure the ecological and economic benefits of these interventions. UNEP has lagged, however, in developing a systematic strategy for quantifying the health benefits of its work.

Quantification of the health benefits of environmental interventions will strengthen national and international agencies’ ability to educate policy makers and the global public that these interventions have real value for people in their daily lives.

## Recommendations

Our main recommendation is that national governments and international agencies develop metrics and strategies and for quantifying the health benefits of interventions against climate change, pollution, and biodiversity loss. We recommended that agencies deploy health benefit assessments in parallel with assessments of environmental and economic benefits.

The metrics of disease, disability, and premature death that have developed by the Global Burden of Disease (GBD) study may provide a useful starting point for health benefit assessment [14,121,122].These metrics are well-validated. They are increasingly used by the World Health Organization and many ministries of health [123]. Like all metrics, they have shortcomings. They fail, for example, to capture some of the broader aspects of human health and well-being such as cognitive function and happiness that are encompassed in the WHO definition of health [124].

Nonetheless, the GBD metrics have the great advantage that they are highly standardized. They enable comparisons of disease burden within and between countries, and because they now span several decades, they enable examination of time trends in patterns of disease and death. They are used in many countries to quantify the positive and negative health impacts of many interventions and to set funding priorities.

Failure to quantify the negative health impacts of environmental degradation and the health benefits of environmental improvements is a lost opportunity. It means that health benefits are not considered in many policy decisions.

We recommend that all environmental intervention projects measure health benefits using the key GBD metrics of Years of Life Lost (YLL) Years lived with Disability (YLD) and Disability-Adjusted Life Years (DALYs) [14,121,122]. Agencies may wish to add additional tools and metrics to gauge human health benefits and detriments. These could include risk factor analyses, environmental hazard analyses, nutritional assessments, the identification of determinants, vulnerability and adaptation measurements, tools to identify inequities and disparities in health, health impact assessments, and the seascape change and landscape modeling. However, the deployment of GBD-based methodologies should not be delayed while other indicators are being developed.

Incorporation of health metrics into assessments of environmental restoration projects will require that environmental scientists work with health scientists and that agencies establish integrated data collection and information management systems that systematically collect health data and integrate these data with environmental and economic data.

### Specific Recommendations:

**1. Health Benefit Assessments –** Health benefits assessments should be a core component of all program evaluations undertaken by national and international environmental agencies. They should be incorporated into programs and projects from the beginning and undertaken in parallel with ecological and economic assessments. Inclusion of health metrics can improve program planning by anticipating the positive and negative health impacts of proposed interventions and avoiding negative outcomes.
**2. Incorporate Health Assessments into Climate Risk Evaluations** – Prior to the start of each new climate mitigation or adaptation project, it is important to assess a community’s climate-related health risks. Indigenous peoples’ knowledge (traditional ecological knowledge) may serve as an important input to such assessments [125]. Coupled with periodic follow-up health assessments across the project timeline, such evaluation will enable quantification of a project’s positive and negative consequences for human health.
**3. Incorporate Health Assessments into Biodiversity Risk Evaluations –** All programs and projects undertaken by national and international agencies to preserve biological diversity and restore habitats need to include assessments of health impacts. From their beginning, these projects should include strategies for improving community health as integral program components. Indigenous peoples’ knowledge may serve as an important input to such assessments [125]. Particularly important will be quantification of reductions in risk of disease spillover events resulting from biodiversity preservation programs [46].
**4. Build Infrastructure to Support health Benefit Assessments –** To build the capacity necessary to incorporate GBD-based health assessments into environmental improvement projects implemented by national agencies and by international organizations such as UNDP, UNEP and the World Bank, it will be necessary to establish integrated surveillance and information management systems that systematically collect health data and link health assessments to environmental and economic assessments [126,127].

Development of such integrated systems will require building interdisciplinary capacity, cross-disciplinary raining programs, and fostering collaborations among environmental scientists, economists, and health scientists.

Once built, these systems will enable agencies to develop, validate and deploy standardized tools and protocols for holistic data collection, collation, analysis and interpretation [128]. These tools could be used to undertake planning processes that can assist governments to prioritize interventions based on full assessment of economic, health, and environmental performance indicators [129,130].

**5. Comprehensive Planning and Communication –** Health risk assessments will be most effective and will add maximal value to environmental intervention projects when they are incorporated into projects from the earliest stages of planning, incorporate in-depth consultations with affected communities, are periodically updated over the course of a project, and make their findings known to policy makers and the public at project conclusion.
